# Polymorphisms in DNA Repair Gene* XRCC3* and Susceptibility to Breast Cancer in Saudi Females

**DOI:** 10.1155/2016/8721052

**Published:** 2016-01-06

**Authors:** Alaa Mohammed Ali, Huda AbdulKareem, Mohammad Al Anazi, Narasimha Reddy Parine, Jilani Purusottapatnam Shaik, Abdullah Alamri, Akbar Ali Khan Pathan, Arjumand Warsy

**Affiliations:** ^1^Department of Biochemistry, College of Science, King Saud University, Riyadh, Saudi Arabia; ^2^Comprehensive Cancer Center, Department of Women's Imaging, King Fahad Medical City, Riyadh, Saudi Arabia; ^3^Genome Research Chair, Department of Biochemistry, College of Science, King Saud University, Riyadh, Saudi Arabia; ^4^Department of Biochemistry, Center for Science and Medical Studies for Girls, College of Science, King Saud University, P.O. Box 22452, Riyadh 11495, Saudi Arabia

## Abstract

We investigated three common polymorphisms (SNPs) in the XRCC3 gene (rs861539, rs1799794, and rs1799796) in 143 Saudi females suffering from breast cancer (median age = 51.4 years) and 145 age matched normal healthy controls. DNA was extracted from whole blood and genotyping was conducted using PCR-RFLP. rs1799794 showed significant association, where AA and AA+AG occurred at a significantly higher frequency in the cancer patients compared to the control group (OR: 28.1; 95% CI: 3.76–21.12; *χ* ^2^: 22.82; *p* < 0.0001). The G allele was protective and presented with a dominant model. The genotype and allele frequencies of rs861539 C>T and rs1799796 A>G did not show a significant difference when the results in the patients and controls were compared. However, the frequency of rs1799796 differed significantly in patients with different age of diagnosis, tumor grade, and ER and HER2 status. The wild type A allele occurred at a higher frequency in the ER− and HER2− group. Our results among Saudis suggest that some variations in XRCC3 may contribute to breast cancer susceptibility. In conclusion, the results obtained during this study suggest that rs1799794 in XRCC3 shows strong association with breast cancer development in Saudi females.

## 1. Introduction

Damage induced by endogenous and exogenous factors affects the integrity and stability of DNA but is constantly and effectively corrected by the DNA repair pathways. Some serious mutations in these genes are shown to result in disorders such as* Xeroderma pigmentosa*; however, a wide variety of common polymorphisms are reported to be linked to mild defects in DNA repair which may predispose a person to the development of different forms of cancer [1–3]. Extensive studies have been conducted among different populations to identify such polymorphisms, but the results from different populations are controversial [4, 5]. Several DNA repair pathways are functional and repair different types of damage, where the double strand breaks (DSB) are corrected by either homologous recombination repair (HRR) or nonhomologous end-joining pathways [6]. The cell's susceptibility to DNA damage and its ability to repair this damage are important for cancer induction, promotion, and progression. Among the different DNA repair genes of the HRR, the X-ray repair complementing defective repair in Chinese hamster cells 3 (*XRCC3*) has been the subject of considerable investigation.* XRCC2* and* XRCC3* genes are structurally and functionally related to RAD51 which plays an important role in homologous recombination, a process, if defective, frequently involved in cancer transformation [7].

X-ray repair complementing defective repair in Chinese hamster cells 3 (XRCC3) is a member of the RecA/Rad51-related protein family that participates in HRR, maintaining chromosome stability and participating in DNA repair. XRCC3 is one of the protein components involved in the HRR pathway and Liu et al. [8] showed that XRCC3 interacts directly with RAD51 and may cooperate with it during recombinational repair mechanisms. Several polymorphisms have been identified in this gene where some of the resulting genetic variants may alter the capacity of DNA repair mechanism and hence may be associated with increased or decreased susceptibility to tumor genesis [9]. Genetic polymorphisms in HRR genes that can lead to protein haploinsufficiency are generally associated with increased cancer risk. Though the* XRCC3* gene is a highly suspected candidate gene for cancer susceptibility, several association studies on the* XRCC3* polymorphisms in cancer have reported conflicting results [10].

The previous published data on the association between* XRCC3* Thr241Met, A4541G, and A17893G polymorphisms and breast cancer risk remains largely controversial, and population related differences in association are reported frequently. Kuschel et al. [2] performed genetic association studies in a population-based breast cancer case-control study and analyzed polymorphisms in 7 genes involved in DNA repair. Genotype frequencies differed between cases and controls for 2 polymorphisms in the* XRCC3* gene. However, most subsequent studies on Caucasians failed to confirm this association [10, 11], though a few studies did report that* XRCC3* Thr241Met is related to an increased risk of breast cancer. Several meta-analyses using pooled data were performed and some showed a small but significant increase in cancer risk [12–15].

In Saudi Arabia, as in several other countries of the world, breast cancer is the most common cancer in females [16]. Though the incidence of breast cancer is lower than in Western countries, it ranks highest, amongst all the malignancies seen among Saudi females [16]. Interest in the molecular genetics of cancer among Saudis has gained momentum over the last few years and different DNA repair genes are the focus of several studies. With this background we initiated this case-control study on Saudi breast cancer patients and investigated three commonly reported* XRCC3* polymorphisms (Thr241Met c.722 (rs861539), c.562-14 (rs1799796), and c.316 (rs1799794)) in the patients in comparison with the healthy controls.

## 2. Material and Methods

### 2.1. Study Population

This study was approved by the Institutional Review Board (IRB) of King Fahad Hospital (KFH). Written informed consent was obtained from all participants. This was conducted as a case-control study involving 143 females (median age = 51.3 ± 10 yrs) suffering from breast cancer and 145 age matched normal healthy controls, all of Saudi Arabian ethnicity. The breast cancer patients were attending the outpatient clinics of the clinical coinvestigators (KFH) in Riyadh, Saudi Arabia, and the controls were also attending KFH for minor illnesses. They were recruited following physical examinations after diagnostic exclusion of cancer or history of cancer and cancer-related diseases. Demographic data, age at diagnosis, tumor grade and receptor status of estrogen receptor (ER), progesterone receptor (PR), and human epidermal growth factor receptor 2 (HER2) were recorded.

### 2.2. DNA Extraction

Approximately 3 mL blood samples were collected by venepuncture in ethylene diamine tetra acetic acid (EDTA) containing vacutainer tubes from all subjects enrolled in the study. DNA (genomic) was extracted from blood samples using a QIAamp DNA blood mini kit (Qiagen, Valencia, CA, USA) following the manufacturer's instructions. The extracted DNA was spectrophotometrically quantitated using NanoDrop 8000 (Thermo Scientific, USA), and purity was determined using the standard A260/A280 and A260/A230 ratios.

### 2.3. Genotyping

Three SNPs in* XRCC3* were genotyped using PCR-RFLP. The fragment of interest was amplified with the PCR primers listed in Table 1.

For the PCR, 25 *μ*L PCR mixture was prepared and it contained 20 ng of DNA, 10 pmol of each primer, 0.2 mmol/L of dNTPs, 2 mmol/L of MgCl_2_, and 1 U of Taq DNA polymerase. Thermal cycling conditions used during this study consisted of an initial denaturation step at 95°C for 10 min followed by 35 cycles of 95°C for 45 sec., 60°C for 50 sec., and 72°C for 45 sec., and a final step at 72°C for 10 minutes. All PCR products were subjected to electrophoresis and stored at 4°C until required for further analysis. The fragment size obtained using each primer set is presented in Table 2. The fragments obtained were treated with the specific restriction endonuclease, that is,* FokI* for rs1799794,* PvuII* for rs1799796, and* NlaIII* for rs861539. The concentrations of each enzyme used were as follows: 1 U of the enzyme* FokI* with BSA (1 *μ*L) and 5 *μ*L PCR products; 0.5 *μ*L* NlaIII* with 0.5 *μ*L BSA and 1 *μ*L buffer 4 used with 5 *μ*L PCR product; and 0.2 *μ*L* PvuII* and 1 *μ*L of buffer X2 NEB with 5 *μ*L of the PCR product. For* FokI* and* NlaIII*, digestion conditions were as follows: incubation at 37°C for 3 hours followed by incubation at 65°C for 20 min., while for* PvuII*, the* PCR* product was incubated at 37°C for 3 hours. After digestion, the digestion products were subjected to electrophoresis in 3% agarose gel, and the size of the fragments obtained for each genotype is presented in Table 2.

Five percent of the samples were randomly selected and subjected to sequencing analysis as a quality control measure for verification of genotyping procedures. The results were reproducible without any discrepancies.

### 2.4. Statistical Analysis

Genotypes were assigned to each sample, and genotype and allelic frequencies were manually calculated and checked for deviation from the Hardy-Weinberg equilibrium (HWE) (https://ihg.gsf.de/cgi-bin/hw/hwa1.pl). The control and patients obeyed HWE (*p* > 0.05). The genotype and allele frequencies in cases and controls were compared using the chi square test and odds ratios (OR), and 95% confidence intervals (CI) were calculated by Fisher's exact test (two-tailed). Patients were grouped based on the age of diagnosis (<48 and >48), tumor grade (grades I, II, III, and IV), and hormone receptor status (ER+/ER−; PR+/PR−; and HER2+/HER2−). The genotype and allele frequencies were calculated in each group and compared using SPSS 18.0 for Windows. A *p* value of ≤0.05 was considered statistically significant.

## 3. Results

The age of the female patients suffering from breast cancer ranged from 28 to 79 years and that of the normal control ranged from 28 to 68 years. The age of diagnosis varied, but the majority were diagnosed above the age of 48 years. The ER, PR, and HER status were used to group the females as positive (+) or negative (−) for the receptor and the results are presented in Table 3. The tumor grade was assessed and the majority of the patients were in Grade II or III. 27 patients were triple negative and 40 were triple positive.

The frequency of the genotypes and alleles of the three SNPs was calculated in the total breast patients and control groups and the results are presented in Table 4. One of the SNPs showed association, where the genotype and allele frequencies of rs1799794 (A>G) showed a significant difference when the results in the patients and controls were compared.

The genotype and allele frequencies of the three SNPs in breast cancer patients were also compared between the patients grouped on the basis of age of diagnosis, tumor grade, and ER, PR, and HER status. The genotype and allele frequencies of rs1799794 did not show any difference between the ER+/ER−, PR+/PR−, and HER+/HER− patients, patients with different tumor grades and patients with different ages of diagnosis (results not shown). Most significant differences were seen in the frequency of rs1799796 in patients with different age of diagnosis, different tumor grades, and different ER and HER2 status. Only the results that were significantly different are presented in Table 5. Between PR+ and PR−, there were no significant differences in the genotype and allele frequency of rs1799796 (results not presented).

Of the 143 breast cancer patients, there were 27 (18.9%) triple negative (ER−, PR−, and HER2−) patients. The genotype frequencies of the three studied SNPs were calculated in the triple negative group and compared to the results obtained in the normal control. Genotype and allele frequencies of rs861539 and rs1799796 were not different in the patients and control group; however, significant differences were seen in the frequencies of rs1799794 in the triple negative cancer patients and control group. Only the results showing significant differences are presented in Table 6.

## 4. Discussion

It is well established that an individual's capacity or risk toward developing cancer can be altered by genetic variations in DNA repair genes [17]. Polymorphisms that lead to protein haploinsufficiency are associated with an increased risk of cancer development. Studies conducted over the past decade have identified such variations in a number of DNA repair genes, which are associated with either an increased susceptibility or an increased resistance to the development of cancer since they may modify DNA repair capacity. These repair genes have a pivotal role in maintaining genomic stability through different pathways and their correct functioning is important for genetic stability. Among the many genes that have been studied in recent years,* XRCC3* has been implicated as one of the candidates. It contributes to important DNA repair mechanisms and plays a role in the repair of double strand breaks induced by a variety of external and internal factors, including ionizing radiation, alkylating agents, and reactive oxygen species. Studies in humans and mice with* XRCC3* gene disruption confirm that these responses are likely to contribute to cancer induction and/or progression [18–20]. It is emphasized that the study of such variations may help in understanding the aetiology of cancer.

Studies in several populations have shown that* XRCC3* has several SNPs which exhibit polymorphism and that different populations differ significantly in the genotype and allele frequencies of these SNPs [21]. Studies conducted on different types of cancer show several contradictory results, where some studies have shown an association between some SNPs in* XRCC3* and a risk of colorectal cancer, gastric cancer, breast cancer, colorectal cancer, lung cancer, glioma and meningioma, liver cancer, or head and neck cancer [3, 22–29], while other studies have failed to show any association [30, 31]. Sliwinski et al. [32] suggested that these polymorphisms in the* XRCC3* gene might be used as a predictive factor of precancerous lesion for head and neck cancer in a Polish population. In this respect a few studies related to different polymorphisms in* XRCC3* and their interaction with different cancers are listed in Table 6, which also shows several contradictory findings.

In this study on breast cancer patients and healthy controls, all the three studied SNPs exhibited polymorphism in the normal Saudi population and cancer patients. All three genotypes were identified for each SNP and the results suggested that some variations in XRCC3 may contribute to breast cancer susceptibility. Significant association was shown by rs1799794, an A to G transition in the 5′UTR, where the mutant G allele was highly protective against breast cancer (OR = 0.48; 95% CI = 0.32–0.74; *χ* ^2^ = 11.73; *p* < 0.0001). The protective effect was more obvious in the homozygotes for the G allele (OR = 0.48; 95% CI = 0.32–0.74; *χ* ^2^ = 11.73; *p* < 0.0001). This result is in agreement with several reports that have shown the G allele to be protective against lung cancer and non-small-cell lung cancer in Chinese [33, 34] and esophageal and gastric cancer in Germans [35], and it was recently suggested that it may have a protective effect against late adverse effects induced by radiotherapy [36]. In a report from Jordan, the G allele was shown to be associated with breast cancer [37], while no association was reported from the UK population [2].

The rs1799794 also showed a significant association with the triple negative cancer, since the G allele was seen to be significantly protective while the wild type A allele was significantly predisposing to breast cancer development in patients who were triple negative. Hence,* XRCC3* rs1799794 AA is a potential predictive marker for triple negative breast cancer in Saudi women and further investigations in other populations are warranted for universal application in cancer detection and prediction.

We also evaluated rs1799794 as a predictive marker for early disease onset, disease severity, and association with the hormone receptor (ER, PR, and HER2) status, but no association was observed. Hence, it can be stated that rs1799794 is associated with breast cancer and triple negative breast cancer in Saudis, but not with disease onset or severity.

No association was observed between rs861539 and breast cancer in Saudis. This is a C>T transition that results in the substitution of Thr241Met and has been implicated as a potential predictive marker for breast cancer in the Taiwanese population [38]. Among the Polish population, this SNP has been associated with cancer progression and grading. It has also been shown to play a role in increasing risk of breast cancer in the British [2] and Taiwanese population [38], colorectal cancer in Polish population [34], and breast and lung cancer in Taiwanese population [38, 39]. Several other studies and meta-analyses have confirmed an association between the* XRCC3* Thr241Met polymorphism and it is suggested that this may be involved in modifying the risk of cancer [40]. Interestingly some studies show that it may have a protective effect [4, 41], while still others failed to show any association with lung cancer in the Danish [42], breast cancer in the Polish [3], Belgian [43], and Jordanian populations [37], and ovarian cancer in a meta-analysis including Caucasian, Asian, and African populations [44]. During our analysis, no differences were found in the frequency of this SNPs when the patients were separated into groups on the basis of the age of diagnosis, tumor grade, and receptor (ER, PR, and HER2) status. Hence, rs861539 cannot be considered as a predictive marker for breast cancer in Saudis.

Finally, rs1799796, an A>G transition in the* XRCC3*, was studied and the genotypes and alleles failed to show any association with breast cancer in the Saudis. Several studies have investigated this mutation in different cancers and the results are contradictory even in patients suffering from the same type of cancer in different populations, as shown in Table 7. A study from the British population showed that rs1799796 decreases the risk of breast cancer [2] while another study from Belgium reported an increased risk associated with this SNP in BRCA1 and BRCA2 carriers [43]. More recently, a significant association with ovarian cancer was confirmed in a meta-analysis involving Caucasian, Asian, and African populations [44]. On the other hand, studies on ovarian cancer from the British population [45], lung cancer in Danish population [42], bladder and breast cancer in American population [46, 47], and prostate and urinary bladder cancer in Indians [30, 48] failed to show any association with this SNP. However, when we grouped our cancer patients on the bases of age of disease onset, tumor grade, and hormone receptor (ER, PR, and HER2) status, several interesting associations were observed. The A allele was found to predispose to breast cancer at a younger age, where the patients diagnosed at age <48 years had a significantly higher frequency compared to those who developed cancer at an age later than 48 years. The A allele also occurred at a significantly higher frequency in patients suffering from tumor grade III compared to tumor grade II, suggesting its involvement in disease severity. It also occurred at a significantly higher frequency in ER− and HER2− females compared to their ER+ and HER2+ counterparts. Hence, we suggest that rs1799796 may be considered as a disease severity marker.

In conclusion, it is obvious that populations differ in the genotype and allele frequencies of different SNPs and also differ in the extent and nature of association of a genotype or allele with disease development. Association studies play an important role in identification of genetic markers that may help in presymptomatic diagnosis of a disease state, including cancer. In addition, therapeutic measure may be directed towards those SNPs that influence gene expression of the respected gene. One drawback of such studies is that the frequencies of the SNP alleles show significant differences between different populations, even in the control groups, hence making it mandatory that studies of this nature need to be performed for each individual population, in an attempt to identify “population specific markers.” In addition, such studies will contribute towards the development of the field of “personalized medicine,” with individualized treatment strategies.

## Figures and Tables

**Table 1:** Primers used for amplification of SNPs in *XRCC3*.

SNPs in *XRCC3*	Primers	
Thr241Met c.722 (rs861539)	F	5′-GCTGTCTCGGGGCATGGCTC-3′
	R	3′-TTTAGCCAGGATGCGGAAGC-5′

c.316 (rs1799794)	F	5′-TGAGGCGCCTAATCAGC-3′
	R	3′-CGCTGCTTGACACAGTCCA-5′

c.562-14 (rs1799796)	F	5′-GACACCTCTACAGAGGACG-3′
	R	3′-CTGTGCCTAACCATCGAGAA-5′

**Table 2:** The size of PCR products and fragment generated upon treatment with restriction enzyme for the three SNPs in *XRCC3*.

*XRCC3* SNP	PCR product size (bp)	RE used	Genotype	Fragment sizes (bp) after RE treatment
*Thr241Met* (rs861539)	231	*NlaIII*	C>T	CC: 231, 12 bp CT: 231, 107, 112 & 12 bp TT: 112, 107 & 12 bp

rs1799794	293	*FokI*	A>G	AA: 100 & 193 bp AG: 100, 193 & 293 bp GG: 293 bp

rs1799796	650	*PvuII*	A>G	AA: 650 bp AG: 283, 367 & 650 bp GG: 283 & 376 bp

RE: restriction endonuclease; SNP: single nucleotide polymorphism; PCR: polymerase chain reaction; bp: base pair.

**Table 3:** Demographic data, tumor grade, and receptor status of the breast cancer patients, investigated during this study.

	Average age (years)	Tumor grade (number of patients)	ER status (number of patients)	PR status (number of patients)	HER2 status (number of patients)
Cancer patients	Group 1: <48 = 72 Group 2: >48 = 71	I = 18 II = 71 III = 45 IV = 9	ER+ = 55 ER− = 88	PR+ = 59 PR− = 84	HER2+ = 71 HER2− = 69

**Table 4:** Genotype and allele frequencies of studied SNPs in breast cancer patients and controls.

	Cancer (total = 143)	Control (total = 145)	Breast cancer versus control			*p* value
	Number (%)	Number (%)	OR	95% CI	*χ* ^2^	
*XRCC3 Thr241Met* rs861539 C>T, genotype frequency						
CC	43 (30.07)	32 (22.07)	Ref.			
CT	73 (51.05)	78 (53.79)	0.7	0.4–1.25	1.41	0.236
TT	27 (18.89)	35 (24.14)	0.59	0.3–1.16	2.35	0.125
CT+TT	100 (69.93)	113 (77.93)	0.67	0.4–1.15	2.11	0.146
CT+CC	116 (81.11)	110 (75.86)	1.35	0.8–2.39	1.11	0.292

Alleles frequency						
C	159 (55.59)	142 (48.97)	Ref.			
T	127 (44.41)	148 (51.03)	0.77	0.6–1.08	2.29	0.1

*XRCC3* rs1799794 A>G, genotype frequency						
AA	102 (71.33)	93 (64.14)	Ref.			
AG	40 (27.97)	28 (19.3)	1.3	0.7–2.28	0.86	0.353
GG	1 (0.007)	24 (16.55)	0.03	0.005–0.28	20.77	<0.0001
AG+GG	41 (28.67)	52 (35.86)	0.72	0.44–1.18	1.7	0.191
AA+AG	142 (99.3)	121 (83.44)	28.1	3.76–21.12	22.82	<0.0001

Alleles frequency						
A	244 (85.33)	214 (73.74)	Ref.			
G	42 (14.68)	76 (26.2)	0.48	0.32–0.74	11.73	<0.0001

*XRCC3* rs1799796 A>G, genotype frequency						
AA	59 (41.26)	61 (42.07)	Ref.			
AG	53 (37.06)	55 (37.93)	0.99	0.59–1.67	0.00	0.988
GG	31 (21.67)	29 (20.0)	1.10	0.59–2.05	0.10	0.751
AG+GG	84 (58.74)	84 (57.9)	0.9	0.51–1.59	0.12	0.726
AA+AG	112 (78.32)	116 (80.0)	1.03	0.65–1.65	0.02	0.889

Alleles frequency						
A	171 (59.79)	177 (61.03)	Ref.			
G	115 (40.21)	113 (38.96)	1.05	0.75–1.47	0.09	0.76

OR = odds ratio; 95% CI = 95% confidence interval; *χ* ^2^ = chi square. SNP = single nucleotide polymorphism; Ref.: reference.

**Table 5:** Comparison of the genotype and allele frequencies of the three SNPs in breast cancer patients, grouped on the basis of age of diagnosis, tumor grade, and ER and HER status (only results that have a statistically significant difference between the compared groups are shown).

Grouping parameter	Group		SNP statistics			
Age of diagnosis *rs1799796 (A>G)* Genotype	<48 yrs frequency (%)	>48 yrs frequency (%)	OR	CI	*χ* ^2^	*p* value
AA	51.39	30.98	0.32	0.13–0.8	6.05	0.014
AG	33.33	40.84	2.03	0.95–4.3	3.42	0.06
GG	15.28	28.18	3.06	1.2–7.6	6.05	0.013
A	68.06	51.41	0.49	0.3–0.80	8.24	0.005
G	31.94	48.59	2.01			

Tumour grade *rs1799796 (A>G)* Genotype	II frequency (%)	III frequency (%)	OR	CI	*χ* ^2^	*p* value

AA	35.71	56.82	5.66	1.47–21.8	7.29	0.007
AG	40.00	36.36	0.57	0.25–1.3	1.77	0.18
GG	24.29	6.82	0.17	0.05–0.68	7.29	0.007
A	55.71	75.00	2.38	1.32–4.29	8.64	0.003
G	44.29	25.00	0.41			

ER status *rs1799796 (A>G)* Genotype	ER− frequency (%)	ER+ frequency (%)	OR	CI	*χ* ^2^	*p* value

AA	43.75	36.78	0.36	0.13–0.97	4.24	0.039
AG	41.70	36.78	1.35	0.63–2.88	0.60	0.44
GG	14.65	26.44	2.77	1.03–7.45	4.24	0.04
A	64.58	55.17	0.56	0.34–0.94	4.96	0.026
G	35.42	44.83	1.77			

HER status *rs1799796 (A>G)* Genotype	HER− frequency (%)	HER+ frequency (%)	OR	CI	*χ* ^2^	*p* value

AA	47.89	33.80	0.29	0.11–0.75	6.91	0.009
AG	39.43	35.34	1.26	0.59–2.71	0.37	0.541
GG	12.68	30.88	3.45	1.3–8.8	6.91	0.009
A	67.61	51.47	0.51	0.31–0.83	7.52	0.006
G	32.39	48.53	1.96			

OR = odds ratio; 95% CI = 95% confidence interval; *χ* ^2^ = chi square. SNP = single nucleotide polymorphism.

**Table 6:** Comparison of the genotype and allele frequencies of rs1799794 A>G in triple negative patients compared to the health control group.

	Triple negative cancer (total = 27)	Control (total = 145)	Breast cancer versus control			*p* value
	Number (%)	Number (%)	OR	95% CI	*χ* ^2^	
*XRCC3* rs1799794 A>G, genotype frequency						
AA	7 (25.92)	93 (64.14)	Ref.			
AG	16 (59.26)	28 (19.3)	0.949	0.35–2.58	0.01	0.91
GG	4 (14.81)	24 (16.55)	0.09	0.005–1.52	5.21	0.022
AG+GG	20 (74.07)	52 (35.86)	0.511	0.19–1.35	1.89	0.168
AA+AG	23 (85.19)	121 (83.44)	11.09	0.65–8.05	5.19	0.022

Alleles frequency						
A	30 (55.55)	214 (73.74)	Ref.			
G	24 (44.44)	76 (26.2)	0.35	0.15–0.855	5.7	0.016

OR = odds ratio; 95% CI = 95% confidence interval; *χ* ^2^ = chi square. SNP = single nucleotide polymorphism.

**Table 7:** Association studies of rs1799794 (A/G), rs861539 (C/T), and rs1799796 (A/G) in XRCC3 in different types of cancer and in different populations.

Cancer types	Effect	Number	Population	Ref.
rs1799794 (A/G), association of G allele				
Breast cancer	Protective	143	Saudis	*∗*
Esophageal and gastric	Survival	258	Germans	[35]
Lung cancer	Decreased risk in carriers of G allele	—	Chinese	[33]
Breast cancer risk	Modified in patients carrying BRCA1, BRCA2 mutation	—	Belgian	[43]
Prostate cancer	Gastrointestinal toxicity	698	Spanish	[49]
Non-small-cell lung	Protective effect of G allele	507	Chinese	[34]
Breast cancer	No association		British	[2]
Ovarian cancer	Association	—	Meta-analysis	[44]
Breast cancer	Association	—	Jordanian	[37]

*rs861539 *(C/T), association of T allele				
Breast cancer	No association	143	Saudis	*∗*
Colorectal cancer	Increased risk (association)	100	Polish	[34]
Breast cancer	Increased risk (association)	1826	British	[2]
Bladder cancer	Protective role	214	Italian	[41]
Colorectal cancer	Protective role	128	British	[4]
Lung cancer	No association	272	Danish	[42]
Breast cancer	No association. Association with cancer progression and grading	700	Polish	[3]
Breast cancer	Association		Taiwanese	[38]
Breast cancer	No association		Jordanian	[37]

*rs1799796 (*A/G), association of G allele				
Breast cancer	No association	143	Saudi	*∗*
Bladder cancer	No association	696	American	[46]
Oral premalignant lesions	Strong association (increases risk)	147	American	[50]
Prostate cancer	No association	192	Indian	[48]
Urinary bladder cancer	No association	211	Indian	[30]
Breast cancer	Increases risk in BRCA1, BRCA2	—	Belgian	[43]
Breast cancer	Decreased risk	2205	British	[2]
Breast cancer	No association	1004	American	[47]
Lung cancer	No association	265	Danish	[42]
Ovarian cancer	No association	1600	British	[45]
Ovarian cancer	Association		Meta-analysis	[44]

*∗* = this study.
